# Childhood behaviour problems predict crime and violence in late adolescence: Brazilian and British birth cohort studies

**DOI:** 10.1007/s00127-014-0976-z

**Published:** 2014-10-16

**Authors:** Joseph Murray, Ana M. B. Menezes, Matthew Hickman, Barbara Maughan, Erika Alejandra Giraldo Gallo, Alicia Matijasevich, Helen Gonçalves, Luciana Anselmi, Maria Cecília F. Assunção, Fernando C. Barros, Cesar G. Victora

**Affiliations:** 1 Department of Psychiatry, University of Cambridge, Douglas House, 18b Trumpington Road, Cambridge, CB2 8AH UK; 2 Post-graduate Program in Epidemiology, Federal University of Pelotas, Pelotas, Brazil; 3 School of Social and Community Medicine, University of Bristol, Bristol, UK; 4 MRC Social, Developmental and Genetic Psychiatry Centre, Institute of Psychiatry, King’s College London, London, UK; 5 Department of Preventive Medicine, Faculty of Medicine, University of São Paulo, São Paulo, Brazil; 6 Postgraduate Program in Health and Behavior, Universidade Católica de Pelotas, Pelotas, Brazil

**Keywords:** Conduct problems, Hyperactivity, Crime, Cohort study, Middle-income country, ALSPAC

## Abstract

**Purpose:**

Most children live in low- and middle-income countries (LMICs), many of which have high levels of violence. Research in high-income countries (HICs) shows that childhood behaviour problems are important precursors of crime and violence. Evidence is lacking on whether this is also true in LMICs. This study examines prevalence rates and associations between conduct problems and hyperactivity and crime and violence in Brazil and Britain.

**Methods:**

A comparison was made of birth cohorts in Brazil and Britain, including measures of behaviour problems based on parental report at age 11, and self-reports of crime at age 18 (*N* = 3,618 Brazil; *N* = 4,103 Britain). Confounders were measured in the perinatal period and at age 11 in questionnaires completed by the mother and, in Brazil, searches of police records regarding parental crime.

**Results:**

Conduct problems, hyperactivity and violent crime were more prevalent in Brazil than in Britain, but nonviolent crime was more prevalent in Britain. Sex differences in prevalence rates were larger where behaviours were less common: larger for conduct problems, hyperactivity, and violent crime in Britain, and larger for nonviolent crime in Brazil. Conduct problems and hyperactivity predicted nonviolent and violent crime similarly in both countries; the effects were partly explained by perinatal health factors and childhood family environments.

**Conclusions:**

Conduct problems and hyperactivity are similar precursors of crime and violence across different social settings. Early crime and violence prevention programmes could target these behavioural difficulties and associated risks in LMICs as well as in HICs.

**Electronic supplementary material:**

The online version of this article (doi:10.1007/s00127-014-0976-z) contains supplementary material, which is available to authorized users.

## Introduction

Childhood conduct disorder and attention deficit hyperactivity disorder are important contributors to the global burden of disease [1]. Prominent theories suggest that these behaviour problems play an important role in the development of interpersonal violence [2, 3], which is itself a major global cause of healthy life years lost, particularly in low- and middle-income countries (LMICs) in Africa and Latin America [4, 5]. Conduct problems might increase the risk for participation in crime because they contribute to poor psychosocial functioning, for example poor relationships with parents and peers, poor educational performance, or drug use [2]. Prospective studies in high-income countries (HICs) show robust associations between conduct problems, crime and violence [6–10], but less consistent associations with hyperactivity [6–12] when controlling for prior social and biological risk factors. The effects of behaviour problems on crime appear similar for males and females [6, 9], or slightly stronger for males [7, 11].

Previous studies compared the effects of childhood behaviour problems on crime between the US, Canada, Britain, New Zealand, and Australia [7, 13, 14]. All found similarity in the effects of conduct problems [7], impulsivity [13, 14], concentration problems [13], and hyperactivity [7] on crime between sites. However, we are not aware of any previous comparison between contexts as different as Brazil and Britain. Although 90 % per cent of the world’s 2.2 billion children and adolescents live in LMICs [15], and many LMICs have high rates of violence [16], it is not known whether childhood behaviour problems have similar effects on crime and violence in LMICs.

We examined associations between conduct problems and hyperactivity at age 11, and nonviolent and violent crime at age 18 in a large, prospective study of a population sample in Pelotas, Brazil, and compared results with a well-matched study in Britain (ALSPAC). Pelotas is a relatively poor city in a relatively rich state of southern Brazil. When crime data were collected for this study in 2011, there were 18.9 homicides in Pelotas per 100,000 population, lower than the national rate of 27.1, but considerably higher than in England and Wales (1.1) and Avon and Somerset (1.1), where the British study is set. This is the first major longitudinal survey of self-reported offending in Brazil [16] and, to our knowledge, the first comparison of prospective risk factors for crime between any LMIC and a HIC. The study had three main questions:

1. What is the prevalence of conduct problems and hyperactivity at age 11, and self-reported crime and violence at age 18 in Pelotas, Brazil, and how do prevalence rates compare with ALSPAC, Britain?
2. Do conduct problems and hyperactivity at age 11 predict increased risk of crime and violence in both Brazil and Britain, and are these associations similar for females and males?
3. Do conduct problems and hyperactivity predict crime and violence independently of confounders in Brazil and Britain?

## Method

### 1993 Pelotas Birth Cohort Study, Brazil

The 1993 Pelotas Birth Cohort Study is an ongoing population-based study designed to investigate the effects of a wide range of influences on health and development. Pelotas is a city located in the extreme south of Brazil, with an estimated population of 345,179 inhabitants, 93 % of whom live in the urban area. All births occurring in the five maternity clinics in the town were monitored in 1993 (99 % of births in Pelotas occurred in hospital). For the 5,265 children born alive, only 16 mothers could not be interviewed or refused to participate in the study. The 5,249 newborns, whose mothers lived in the urban area, were included in the cohort. The detailed methodology of this study can be found elsewhere [17]. During the perinatal study, mothers were interviewed to collect demographic, health and socioeconomic information about the family. Follow-up home visits were conducted in 2004–2005 (age 11) and in clinic sessions in 2011–2012 (age 18) [18]. The perinatal study and each follow-up were approved by the Research Ethics Committee of the Federal University of Pelotas School of Medicine. After being informed of the details of the study, participants signed a term of informed consent.

### Avon Longitudinal Study of Parents and Children (ALSPAC), Britain

ALSPAC is a separate, ongoing population-based study in Britain. ALSPAC recruited 14,541 pregnant women residents in Avon, Britain with expected dates of delivery from 1st April 1991 to 31st December 1992; and, from age 7, continued to recruit children born in that area at that time until age 18. The total sample size for analyses using any data collected after the age of seven is 15,247 pregnancies, resulting in 15,458 children. We used data on 14,762 live-born singleton or twin children; triplets and quads were excluded for reasons of confidentiality. The detailed methodology of ALSPAC can be found elsewhere [19, 20] and the study website contains details of all the data that is available through a fully searchable data dictionary (http://www.bris.ac.uk/alspac/researchers/data-access/data-dictionary/). When compared to 1991 National Census Data, the ALSPAC sample was found to be similar to the UK population as a whole, but had a slightly higher proportion of married or cohabiting mothers and families who were owner occupiers, and (consistent with the area where the study is based), a smaller proportion of mothers from ethnic minorities (2.2 versus 7.6 %) [20]. When cohort members were 11 years old, mothers completed questionnaires about the children. When cohort members were 18 years old, adolescents participated in focus clinic sessions. Ethical approval for the study was obtained from the ALSPAC Ethics and Law Committee and the Local Research Ethics Committees.

## Measures

### Behaviour problems at age 11

When children were 11 years old, parents (usually mothers) completed the Strengths and Difficulties Questionnaire (SDQ) for 4,423 children in Pelotas and 7,307 children in ALSPAC. The SDQ is a screening questionnaire that assesses child mental health symptoms in the previous 6 months. It includes sub-scales measuring two types of child behaviour problems: conduct problems (symptoms of oppositional defiant and conduct disorders) and hyperactivity (symptoms of inattention and hyperactivity disorders). The SDQ was developed by Goodman [21] and validated in Brazil by Fleitlich-Bilyk and Goodman [22]. A previous study in Pelotas compared the SDQ with a diagnostic instrument [Development and Well-Being Assessment (DAWBA)]. In relation to diagnoses on DAWBA as the gold standard, the psychometric properties of the SDQ were 78.2 % sensitivity, 70.4 % specificity, and 74.0 % area under the curve [23]. The same cut-points were used in Pelotas and ALSPAC to identify “abnormal” levels of conduct problems (>3) and hyperactivity (>6).

### Crime and violence at age 18

A confidential self-reported crime questionnaire, originally developed in the Edinburgh Study of Youth Transitions and Crime [24], was completed by 4,102 adolescents in ALSPAC clinic sessions at age 18. Thirteen questions from this instrument were then included in the confidential questionnaire in the Pelotas study clinic sessions at age 18, referring to crimes committed by the adolescents in the previous 12 months. For the Pelotas study, questions were first translated into Brazilian Portuguese, then pilot tested among adolescent offenders in Pelotas (in a young offenders’ institution) and among adolescents in the community (in a public health clinic), adjusted by bilingual researchers, further pilot tested, and then back translated into English. Due to a printing error, the first 325 questionnaires (8 % of 4,106 participants at age 18) in Pelotas were not usable. The current analyses of criminal behaviour in Pelotas included the vast majority of participants (*N* = 3,618) with complete crime data from these questionnaires. The Pelotas sub-sample without crime data is extremely similar to the majority with valid crime data on all perinatal characteristics (see the Online Supplement, Table S1).

We used two summary crime variables as outcomes in our analyses: (1) reported at least one of nine types of nonviolent crimes: stole from shops/stores, damaged property, stole from vehicle, stole vehicle, sold drug, burgled, sold stolen good, arson, stole from person without threat/force; (2) reported at least one of four types of violent crime: stole from person with threat/force, assault, carried a weapon for fights or self-defence, used weapon. Police and justice system records were also searched in Pelotas. In the main analyses we use only self-reported crime data, to maximise comparability with the British study, but we note here that, in Pelotas, the association between self-reported crime and officially recorded crime at age 18 was strong (risk ratio = 4.4 for nonviolent crime and 5.2 for violent crime).

### Confounding variables

Numerous biological, psychological and social variables from pregnancy through late childhood predict the development of antisocial behaviour [6, 12, 25–27]. In both studies, we included confounders measured with mothers in the perinatal period, and indicators of parental crime and mental health up to age 11; variables were dichotomised to maximise comparability between studies. The following perinatal characteristics were measured in both studies: unplanned pregnancy (yes/no), mother ever smoked during pregnancy (yes/no), mother used alcohol during pregnancy (yes/no), maternal urinary infection during pregnancy (yes/no), intrauterine growth restriction (yes/no; referring to <10th percentile/≥10th percentile for gestational age and gender, according to the reference curve developed by Kramer et al. [28]), premature birth <37 weeks (yes/no).

The following socio-demographic characteristics were measured in both studies in the perinatal period: maternal age (<20/≥20 years), low maternal education (yes/no; referring in Pelotas to 0–8 versus ≥9 years of schooling; referring in ALSPAC to qualified up to certificate of secondary qualification level, versus qualified to at least vocational level, O-level, or A-level), marital status (single mother/with partner), three or more siblings (yes/no), family income (lowest quintile/second–fifth quintiles). All health and socio-demographic variables in the perinatal period have been carefully compared between Pelotas and ALSPAC in previous work and related to childhood conduct problems and adolescent violence [29, 30]. No variable was correlated with any other variable more than phi = 0.3.

Parental crime (between the child’s birth and age 11), and maternal mental health were also included as confounding variables in this study. In Pelotas, parental crime was measured by searching state-wide police registries to identify whether the mother or father had committed a crime between the child’s birth and 11th birthday. In ALSPAC, parental crime was measured by asking both mothers and their partners about whether either person had been in trouble with the law/convicted since the previous interview on eight occasions between when children were 8 months and 11 years old. Any indication that either parent had been in trouble with the law from the child’s birth to 11 years was coded as positive for parental crime.

Maternal mental health was measured in Pelotas using the self report questionnaire (SRQ) when children were 11 years. The SRQ measures depression and anxiety and was validated in a Brazilian sample of 485 subjects [31]. We used a cut-off of eight points to classify mothers as having probable minor psychiatric disorders. In ALSPAC, maternal mental health was measured using the 10-item Edinburgh Postnatal Depression Scale (EPDS [32]) at 11 years. We used the recommended cut-off of 13 points to identify mothers with probable depression.

### Statistical analyses

The prevalence of conduct problems, hyperactivity, crime and violence were compared between Pelotas and ALSPAC, and between females and males within each study, using risk ratios and 95 % confidence intervals. Associations between behaviour problems (conduct problems and hyperactivity) and nonviolent and violent crime were also examined using risk ratios and 95 % confidence intervals. To investigate possible differences in risk ratios (interactions) between studies or between sexes, the ratio of risk ratios with 95 % confidence intervals was calculated [33]. To calculate adjusted risk ratios, we used Poisson regression with robust standard errors, as proposed by Barros and Hirakata [34].

Comparisons between countries can be conceptualised in different ways. Our use of risk ratios to compare associations between countries has a particular meaning, which can be illustrated in an example of comparing sex differences in violence between countries. Imagine in Country One, the prevalence of violence is 10 % among males and 5 % among females; this equals a male–female risk ratio of 2.0 (10 ÷ 5 %), and a risk difference between males and females of 5 % (10 − 5 %). In Country Two, the prevalence of violence might be 25 % among males and 20 % among females, which equals a lower risk ratio of 1.25 (25 ÷ 20 %), but the same risk difference of 5 % (25 − 20 %). In this hypothetical example, the risk ratio in Country One is larger than in Country Two, although the risk difference is the same in both countries. Both findings, based on risk ratios and risk differences, are correct, but conceptualise the comparison between countries (and sexes) in different ways. Thus, it is important to bear in mind that our cross-national comparisons are based on risk ratios, and we conceptualise a sex difference of 10 versus 5 % in one country as larger than a difference of 25 versus 20 % in another country.

In Pelotas, participants with valid crime data at age 18 had very similar perinatal characteristics compared with participants without crime data; however, this was not true in ALSPAC (see Online Supplement Table S1). To reduce bias caused by missing data, we estimated associations between childhood behavioural problems and adolescent crime using multiple imputation for missing data in both studies. Fifty data sets (each with 2,645 females and 2,603 males in Pelotas, and 5,937 females and 6,242 males in ALSPAC) were created by imputing missing predictor and outcome data using the mi impute-chained command in STATA 12.1. All variables in the multivariate models (crime at 18, age at crime measurement, conduct problems and hyperactivity at 11, and confounding variables) were used in the imputation process. A minimum requirement for inclusion in the multiple imputation analyses was that at least half of the confounding variables were valid or childhood behaviour data were valid or crime data were valid. Logistic regression was used to impute binary variables and OLS regression to impute continuous variables (age). In the main text, results are presented based on multiple imputation; the Online Supplement shows that results based on complete case analyses are very similar.

## Results

Children in Pelotas had about four times higher risk for both conduct problems and hyperactivity measured on the SDQ, compared with children in ALSPAC (Table 1). Interestingly, although the prevalence of self-reported violent crime was higher in Pelotas, the prevalence of nonviolent crime was higher in ALSPAC. There was no difference in the probability of reporting “any crime” between the two sites. Note that the violent behaviour most commonly reported in both studies (assault) was not trivial. The question specified that the assault should have been done with the intention of hurting the victim and excluded fighting with siblings. The majority of adolescents who reported committing assault also stated that they did indeed cause an injury in their most serious fight in the last year (83 % Pelotas; 77 % ALSPAC).

**Table 1:** Prevalence of childhood behaviour problems (age 11) and adolescent crime (age 18) in Pelotas, Brazil and ALSPAC, Britain

	Pelotas females (%)	ALSPAC females (%)	RR (95 % CI)	*p* value	Pelotas males (%)	ALSPAC males (%)	RR (95 % CI)	*p* value
Behaviour problems	*N* = 2,245	*N* = 3,656			*N* = 2,178	*N* = 3,651		
Conduct	28.7	6.0	4.8 (4.1–5.5)	<0.001	33.9	8.5	4.0 (3.5–4.5)	<0.001
Hyperactive	21.3	4.2	5.1 (4.3–6.1)	<0.001	31.4	9.9	3.2 (2.8–3.6)	<0.001
Individual nonviolent Crimes	*N* = 1,847	*N* = 2,252			*N* = 1,771	*N* = 1,760		
Stole from shops/stores	0.7	7.0	0.1 (0.1–0.2)	<0.001	2.4	8.9	0.3 (0.2–0.4)	<0.001
Damaged property	0.6	1.2	0.5 (0.2–1.0)	0.034	4.2	6.4	0.7 (0.5–0.9)	0.005
Stole from vehicle	0.1	0.1	0.6 (0.1–6.7)	0.683	0.7	0.7	0.9 (0.4–2.0)	0.829
Stole vehicle	0.1	0.8	0.1 (0.0–0.5)	<0.001	0.7	2.5	0.3 (0.2–0.5)	<0.001
Sold drug	0.9	2.3	0.4 (0.2–0.7)	<0.001	3.5	6.1	0.6 (0.4–0.8)	<0.001
Burgled	0.1	0.1	0.6 (0.1–6.7)	0.683	0.5	0.4	1.3 (0.5–3.4)	0.625
Sold stolen good	0.7	0.3	2.4(0.9–6.5)	0.065	3.1	1.8	1.7 (1.1–2.6)	0.018
Arson	0.1	0.4	0.3 (0.1–1.4)	0.111	1.0	1.4	0.7 (0.4–1.3)	0.263
Stole from person—no threat/force	0.5	0.5	1.1 (0.5–2.6)	0.813	0.9	0.7	1.3 (0.6–2.8)	0.458
Individual violent crimes	*N* = 1,847	*N* = 2,252			*N* = 1,771	*N* = 1,760		
Assault with intention to injure	7.8	2.4	3.3 (2.4–4.5)	<0.001	18.0	9.2	2.0 (1.6–2.3)	<0.001
Carried weapon	2.2	0.8	2.6 (1.5–4.4)	<0.001	10.2	3.0	3.4 (2.5–4.6)	<0.001
Used weapon	0.2	0.0	N/A	0.027	2.7	0.6	4.8 (2.4–9.4)	<0.001
Stole from person—with threat/force	0.1	0.0	2.4 (0.2–26.9)	0.452	1.0	0.1	8.9 (2.1–38.5)	<0.001
Summary crime variables	*N* = 1,847	*N* = 2,252			*N* = 1,771	*N* = 1,760		
Any crime	10.2	11.2	0.9 (0.8–1.1)	0.325	25.7	23.5	1.1 (1.0–1.2)	0.135
Nonviolent crime	2.7	9.4	0.3 (0.2–0.4)	<0.001	9.7	18.0	0.5 (0.5–0.6)	<0.001
Violent crime	8.9	3.0	3.0 (2.3–4.0)	<0.001	22.6	11.0	2.1 (1.8–2.4)	<0.001

*RR* risk ratio comparing Pelotas and ALSPAC, with ALSPAC as the reference category

Conduct problems and hyperactivity were strongly related to each other in both Pelotas and ALSPAC. In Pelotas, 56.4 % of males with conduct problems had high hyperactivity scores versus 18.5 % of males without conduct problems (RR = 3.0; CI = 2.7–3.4) ; for females equivalent rates were 45.0 versus 11.8 % (RR = 3.8; CI = 3.3–4.5). In ALSPAC, 40.5 % of males with conduct problems had high hyperactivity scores, versus 7.0 % of males without conduct problems (RR = 5.8; CI = 4.8–6.9); for females equivalent rates were 24.6 versus 2.9 % (RR = 8.6; CI = 6.4–11.7).

As one would expect from previous studies, males were more likely than females to show conduct problems, hyperactivity, non-violent, and violent crime, and this was true in both Pelotas and ALSPAC (*p* < 0.001 for all sex comparisons in both studies). However, these sex differences, expressed as male–female ratios, were not equal across sites and types of behaviour. The male–female ratio was larger in Pelotas than in ALSPAC for nonviolent crime (Pelotas = 3.6, CI = 2.6–4.9; ALSPAC = 1.9, CI = 1.6–2.3; *p* for the comparison of ratios = 0.054). However, the male–female ratio was larger in ALSPAC than in Pelotas for violent crime (ALSPAC = 3.7, CI = 2.8–4.9; Pelotas = 2.5, CI = 2.1–3.0; *p* = 0.054), conduct problems (ALSPAC = 1.4, CI = 1.2–1.7; Pelotas = 1.2, CI = 1.1–1.3; *p* = 0.154), and hyperactivity (ALSPAC = 2.4, CI = 2.0–2.9; Pelotas = 1.5, CI = 1.3–1.6; *p* = 0.010). In other words, male–female differences were larger in settings where behaviours were less common––that is, for nonviolent crime in Pelotas, and for violent crime, conduct problems, hyperactivity and in ALSPAC.

### Bivariate associations

**Fig. 1:**
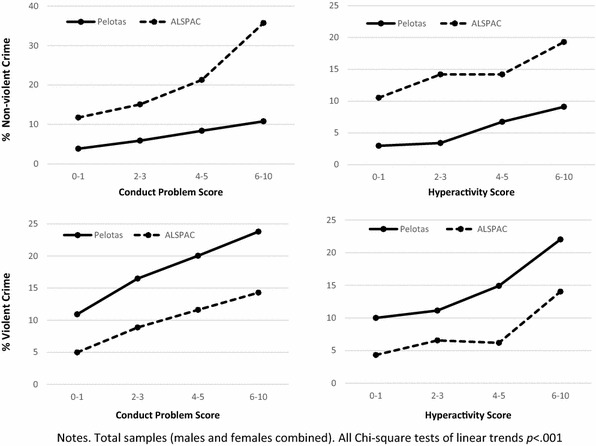
Prevalence of adolescent crime by behaviour problem scores in Pelotas, Brazil and ALSPAC, Britain

In both Pelotas and ALSPAC, children with high conduct problem and high hyperactivity scores were more likely to self-report both nonviolent and violent crime in late adolescence (Tables 2 and 3). To consider how prediction varied with severity of behaviour problems, we also examined the prevalence of crime according to four different levels of behaviour problems. The relationship was almost linear in both studies, between the four levels of conduct problems and hyperactivity, and nonviolent and violent crime (Fig. 1). Note that, at all levels of child behaviour problems, adolescents in Pelotas were more likely to report violent crime than in ALSPAC, and adolescents in ALSPAC were more likely to report nonviolent crime than in Pelotas (Fig. 1). This pattern was true for both females and males when analysed separately, as well as for both sexes pooled (see Online Supplement Table S2).

**Table 2:** Childhood behaviour problems and adolescent nonviolent crime

	Pelotas	Nonviolent crime		ALSPAC	Nonviolent crime	
	*N*	Yes	No	*N*	Yes	No
Females						
Conduct		*p* < 0.001			*p* = 0.009	
Yes	517	5.2 %	94.8 %	97	16.5 %	83.5 %
No	1,284	1.6 %	98.4 %	1,743	8.7 %	91.3 %
Hyperactive		*p* = 0.009			*p* = 0.733	
Yes	397	4.5 %	95.5 %	58	10.3 %	89.7 %
No	1,404	2.1 %	97.9 %	1,782	9.0 %	91.0 %
Males						
Conduct		*p* < 0.001			*p* = 0.001	
Yes	556	13.3 %	86.7 %	85	31.8 %	68.2 %
No	1,127	8.2 %	91.8 %	1,370	17.7 %	82.3 %
Hyperactive		*p* = 0.006			*p* = 0.079	
Yes	522	12.8 %	87.2 %	95	25.3 %	74.7 %
No	1,161	8.5 %	91.5 %	1,360	18.0 %	82.0 %

Row percents

**Table 3:** Childhood behaviour problems and adolescent violent crime

	Pelotas	Violent crime		ALSPAC	Violent crime	
	*N*	Yes	No	*N*	Yes	No
Females						
Conduct		*p* < 0.001			*p* = 0.381	
Yes	517	14.3 %	85.7 %	97	4.1 %	95.9 %
No	1,284	6.6 %	93.4 %	1,743	2.6 %	97.4 %
Hyperactive		*p* < 0.001			*p* = 0.728	
Yes	397	16.1 %	83.9 %	58	3.5 %	96.5 %
No	1,404	6.8 %	93.2 %	1,782	2.7 %	97.3 %
Males						
Conduct		*p* < 0.001			*p* = 0.002	
Yes	556	28.4 %	71.6 %	85	21.2 %	78.8 %
No	1,127	20.7 %	79.3 %	1,370	10.2 %	89.8 %
Hyperactive		*p* < 0.001			*p* < 0.001	
Yes	522	29.3 %	70.7 %	95	22.1 %	77.9 %
No	1,161	20.5 %	79.5 %	1,360	10.1 %	89.9 %

Row percents

All associations between behaviour problems and crime were positive, for girls and boys, in both Pelotas and ALSPAC (Table 4). There was no significant difference in the strength of these associations between Pelotas and ALSPAC (all tests of interaction *p* > 0.05). Comparing associations between females and males within each study, only one interaction out of eight was significant: the association between hyperactivity and violent crime was larger for girls (RR = 2.2, CI = 1.6–3.0) than boys (RR = 1.5, CI = 1.3–1.8) in Pelotas (*p* = 0.027 for interaction).

**Table 4:** Unadjusted associations between childhood behaviour problems and adolescent crime

		Pelotas		ALSPAC		Pelotas–ALSPAC interaction	
		RR (95 % CI)	*p* value	RR (95 % CI)	*p* value	RRR (95 % CI)	*p* value
Females							
Behavioural predictor	Crime outcome						
Conduct	Non-violent	2.7 (1.6–4.5)	<0.001	1.8 (1.2–2.6)	0.002	1.5 (0.8–2.8)	0.223
Hyperactive	Non-violent	1.9 (1.1–3.3)	0.030	1.3 (0.8–2.1)	0.278	1.4 (0.7–3.0)	0.337
Conduct	Violent	1.9 (1.5–2.5)	<0.001	1.8 (1.0–3.2)	0.049	1.1 (0.6–2.1)	0.789
Hyperactive	Violent	2.2 (1.6–3.0)	<0.001	1.8 (0.9–3.5)	0.075	1.2 (0.6–2.5)	0.609
Males							
Behavioural predictor	Crime outcome						
Conduct	Non-violent	1.7 (1.3–2.3)	<0.001	1.7 (1.4–2.1)	<0.001	1.0 (0.7–1.5)	0.961
Hyperactive	Non-violent	1.5 (1.1–2.0)	0.004	1.4 (1.1–1.8)	0.005	1.1 (0.7–1.6)	0.698
Conduct	Violent	1.4 (1.2–1.7)	<0.001	1.9 (1.5–2.4)	<0.001	0.8 (0.6–1.0)	0.097
Hyperactive	Violent	1.5 (1.3–1.8)	<0.001	1.8 (1.4–2.4)	<0.001	0.8 (0.6–1.1)	0.183

Based on 50 data sets using multiple imputation of missing data as described in methods section; *N* = 2,645 females and 2,603 males in Pelotas, and 5,937 females and 6,242 males in ALSPAC

*RR* risk ratio, comparing risk of crime outcome between children with behaviour problem and children without behaviour problem, controlling only for child age in months at time of crime assessment

*CI* confidence interval

RRR = ratio of risk ratios = RR for Pelotas divided by RR for ALSPAC

### Multivariate models

After controlling for confounding variables in multivariate models, associations between childhood behaviour problems and crime were reduced in both Pelotas and ALSPAC (Table 5). Nonetheless, four associations remained significant in Pelotas (with risk ratios ranging from 1.3 to 2.4), and four remained significant in ALSPAC (with risk ratios ranging from 1.4 to 1.6). Again, there was no significant difference in the strength of associations between Pelotas and ALSPAC (all tests of interaction *p* > 0.05). There was also no significant difference in the strength of association comparing females and males within each study (all tests of interaction *p* > 0.05). In sensitivity analyses, we conducted the same analyses using only cases with complete data, rather than using multiple imputation for missing data, and results were very similar (Online Supplement, Table S3).

**Table 5:** Associations between childhood behaviour problems and crime, adjusted for confounders

		Pelotas		ALSPAC		Pelotas–ALSPAC interaction	
		RR (95 % CI)	*p* value	RR (95 % CI)	*p* value	RRR (95 % CI)	*p* value
Females							
Behavioural predictor	Crime outcome						
Conduct	Non-violent	2.4 (1.3–4.3)	0.005	1.6 (1.1–2.3)	0.022	1.5 (0.7–3.1)	0.272
Hyperactive	Non-violent	1.3 (0.7–2.4)	0.434	1.0 (0.6–1.7)	0.872	1.2 (0.5–2.8)	0.612
Conduct	Violent	1.4 (1.0–1.9)	0.050	1.4 (0.8–2.6)	0.272	1.0 (0.5–1.9)	0.930
Hyperactive	Violent	1.8 (1.3–2.5)	0.001	1.5 (0.8–3.0)	0.243	1.2 (0.6–2.5)	0.651
Males							
Behavioural predictor	Crime outcome						
Conduct	Non-violent	1.4 (1.0–2.0)	0.044	1.4 (1.1–1.8)	0.003	1.0 (0.6–1.5)	0.909
Hyperactive	Non-violent	1.2 (0.9–1.7)	0.192	1.2 (0.9–1.5)	0.227	1.1 (0.7–1.6)	0.796
Conduct	Violent	1.2 (1.0–1.5)	0.051	1.4 (1.1–1.8)	0.019	0.9 (0.6–1.2)	0.421
Hyperactive	Violent	1.3 (1.1–1.6)	0.001	1.5 (1.2–2.0)	0.003	0.9 (0.6–1.2)	0.445

Based on 50 data sets using multiple imputation of missing data as described in methods section; *N* = 2,645 females and 2,603 males in Pelotas, and 5,937 females and 6,242 males in ALSPAC

*RR* risk ratio adjusted for conduct and hyperactive problems age 11; unplanned pregnancy, ever smoked in pregnancy, alcohol use in pregnancy, urinary infection in pregnancy, intrauterine growth restriction, premature birth, maternal age, maternal education, marital status, 3+ siblings, low family income, parental crime birth-age 11, maternal mental health age 11, child age in months at time of crime assessment

CI confidence interval

RRR = ratio of adjusted risk ratios = RR for Pelotas divided by RR for ALSPAC

## Discussion

Behaviour problems and violence are major global health problems. In 2010, 5.8-million healthy life years were lost worldwide due to conduct disorder, 0.5 million due to attention deficit hyperactivity disorder, and 25.5 million due to interpersonal violence [35]. In LMICs in the Americas, one-third of all deaths among 15–29-year-olds were caused by interpersonal violence in 2011 [36]. It is important to establish whether key risk factors identified in HICs, such as childhood behaviour problems, influence the development of crime and violence in similar ways in LMIC settings. We found that childhood conduct problems and hyperactivity were similarly associated with crime and violence in two large, population-based, longitudinal studies in Brazil and Britain.

Homicide rates are very high in Brazil compared with HICs such as Britain [16], but there is a lack of reliable data on non-lethal crime and violence in Brazil to compare with other countries. In the first study of its type, we found interesting patterns of self-reported crime: higher rates of violent crime in Brazil than in Britain, but higher rates of nonviolent crime in Britain compared with Brazil. This is consistent with evidence that England and Wales lie in a cluster of Anglo-Saxon countries with high levels of property crime and drug use, but average levels of violence [37]. Brazil may be more similar to a cluster of Eastern European countries, which have high levels of violence but not high levels of property crime or drug use [16, 37]. It has been speculated that, in those countries, increased rates of violence are caused by social inequality, low levels of social control, and widespread material poverty [37], and this may also be true in Brazil [16].

The size of sex differences in behaviour problems and crime varied between our Brazilian and British samples. The male–female ratio was larger in Britain than in Brazil for conduct problems, hyperactivity, and violent crime, but the male–female ratio was larger in Brazil for nonviolent crime. In other words, sex differences were more pronounced in settings where the particular problem behaviour was less common. This is consistent with the most comprehensive analysis to date of sex differences in antisocial behaviour, based on the Dunedin cohort, New Zealand: this showed that the male–female ratio in antisocial behaviour was especially large for less frequent and more serious behaviours in that setting [11].

Although prevalence rates varied substantially between Brazil and Britain, conduct problems and hyperactivity were similarly associated with crime and violence in both sites, speaking to the cross-cultural significance of early behaviour problems in the development of more serious crime and violence. However, in both Brazil and Britain, associations were attenuated when taking into account the co-occurrence of conduct problems and hyperactivity, and other risk factors measured up to age 11. Therefore, conduct problems and hyperactivity predict crime partly because they mark other influences in childhood, including health and socioeconomic factors in the perinatal period, and maternal depression and parental criminality during childhood. Although previous studies have found mixed results for the effects of hyperactivity on crime and violence [6–12], it is notable that, in our samples, hyperactivity was predictive of violence for both females and males in Brazil, and for males in Britain, even after adjustment for confounders.

Our findings suggest that childhood conduct problems and hyperactivity could be significant factors contributing to crime and violence in Brazil as well as in Britain. Early interventions to prevent childhood behaviour problems that can lead to crime should be evaluated in Brazil where rates of violence are particularly high, for example parent-training programmes which have been found effective in other settings [38]. Given the strong association between poverty and adolescent behaviour problems in Pelotas [39], poverty reduction strategies are also needed.

### Strengths and limitations

This study has several important strengths. A major strength is the collection of prospective cohort data on behaviour problems and violence in Brazil. To our knowledge, this is the first Latin American study that has assessed the effects of childhood behaviour problems on later crime and violence. The current study is also novel in its direct comparisons of prospectively measured risk factors for crime between LMIC and HIC settings. Additional strengths are that the study is based on two large, prospective, population-based surveys that are well matched in terms of year of birth, ages at follow-up, and instruments used for key predictor and outcome variables. Also, although most major studies of antisocial behaviour have included only boys, both our Brazilian and British studies included females as well as males. In both Pelotas and ALSPAC, childhood behaviour problems were measured using parental reports and crime was measured using self-reports, reducing the problem of common informant bias, which might otherwise inflate the size of associations. Furthermore, there was a wide range of comparable confounding variables included in both studies. The specificity of results for violent and nonviolent crime suggest that differences in prevalence rates between Brazil and Britain were not an artefact of generic over or under-reporting.

The following limitations of the study should also be considered. There was significant attrition in ALSPAC and missing data were associated with childhood risk factors. Given selective attrition, the prevalence of behaviour problems and crime are probably underestimated in ALSPAC. It was reassuring that we found similar associations between behaviour problems and crime using both complete cases and using multiple imputation for missing data; also some evidence suggests that predictive models are quite robust to missing data [40]. However, multiple imputation cannot guarantee lack of bias caused by selective attrition. Multiple imputation eliminate bias only if enough variables that predict missing values are included in the imputation model. If participants with missing data differ from other participants in ways not reflected by the variables included in the study, the missing at random assumption does not hold and bias cannot be eliminated. Although we included quite an extensive range of predictor variables in this study, we cannot rule out the possibility that results would have been different if there were no missing data.

While corroborative evidence from official records makes it very plausible that rates of violence in Brazil are higher than in Britain, we urge particular caution regarding the extremely high rates of conduct problems and hyperactivity reported for Brazilian children (four times the rates reported in Britain). Although other questionnaire-based studies in Brazil also document very high levels of child behaviour problems [41, 42], there is debate as to whether this reflects overreporting of symptoms by Brazilian parents [42]. It is possible that specific procedures (for example, reading questionnaires to Brazilian parents with less education) or cultural differences in interpreting questionnaire items causes overestimation of children’s behaviour problems in Brazil. If indeed problem behaviours are overreported in Brazilian studies, cut-off points to identify “abnormal” levels of problem behaviour could be raised. However, we believe this methodological issue does not affect our key findings on the associations between childhood behaviour problems and risk for adolescent crime. We found that these relationships were linear across different levels of behaviour problem scores in both Brazil and Britain, suggesting that the specific cut-off point used did not affect the observed strength of associations.

It would have been ideal to include multiple measures of both child behaviour problems (e.g., also diagnostic assessments) and crime (e.g., also official records), which we did not have in both studies. As not all crime is reported to the police, it would have been ideal to also include self-reported criminal behaviours of parents. Finally, while both studies used large community populations, neither used national samples and results reflect each local population.

## Conclusion

In summary, childhood conduct problems and hyperactivity are similar precursors of both nonviolent and violent crime across two very different social contexts. Conduct problems and hyperactivity potentially represent both markers of other childhood risk factors, and possible risk mechanisms increasing the chances of engagement in adolescent crime and violence. These findings speak to the need to evaluate early intervention programmes to reduce childhood behaviour problems in LMICs, as well as in high-income settings.

## Electronic supplementary material

Below is the link to the electronic supplementary material.

Supplementary material 1 (PDF 68 kb)
