# Supplementary material for: Childhood behaviour problems predict crime and violence in late adolescence: Brazilian and British birth cohort studies
Source: Soc Psychiatry Psychiatr Epidemiol. 2014 Oct 16;50(4):579–89. doi: 10.1007/s00127-014-0976-z (PMC4361758; doi:10.1007/s00127-014-0976-z)
Supplement: Supplementary file 1 — Supplementary material 1 (PDF 68 kb) [file 127_2014_976_MOESM1_ESM.pdf]

ONLINE SUPPLEMENT

CHILDHOOD BEHAVIOUR PROBLEMS PREDICT CRIME AND VIOLENCE IN LATE ADOLESCENCE: BRAZILIAN AND BRITISH BIRTH COHORT STUDIES

Joseph Murray, Ana M. B. Menezes, Matthew Hickman, Barbara Maughan, Erika Alejandra Giraldo Gallo, Alicia Matijasevich, Helen Gonçalves, Luciana Anselmi, Maria Cecília F. Assunção, Fernando C. Barros, Cesar G. Victora

PUBLISHED IN SOCIAL PSYCHIATRY AND PSYCHIATRIC EPIDEMIOLOGY

Table S1. Perinatal characteristics by availability of crime data at age 18

	PELOTAS		ALSPAC	
	Valid Crime Data N = 3,618 %	No Crime Data N = 1,631 %	Valid Crime Data N = 4,012 %	No Crime Data N = 10,750 %
**Unplanned pregnancy**	*p*=.447		*p*<.001	
Yes	55.9	57.1	24.61	33.2
No	44.1	42.9	75.4	66.8
**Ever smoked in pregnancy**	*p*=.239		*p*<.001	
Yes	32.9	34.5	15.9	28.0
No	67.1	65.5	84.1	72.0
**Alcohol use in pregnancy**	*p*=.584		*p*=.287	
Yes	5.3	5.0	8.4	7.8
No	94.7	95.0	91.6	92.2
**Urinary infection in pregnancy**	*p*=.818		*p*<.001	
Yes	33.7	33.4	11.8	14.8
No	66.3	66.7	88.2	85.2
**Intrauterine growth restriction**	*p*=.366		*p*=.002	
Yes	90.8	10.0	7.5	9.2
No	9.2	90.0	92.5	90.8
**Preterm birth (< 37weeks)**	*p*=.007		*p*=.853	
Yes	10.6	13.2	5.0	6.8
No	89.4	86.8	95.0	93.2
**Maternal age**	*p*=.731		*p*<.001	
<20	17.6	17.2	1.6	5.8
≥20	82.4	82.8	98.4	94.2
**Maternal education**	*p*=.003		*p*<.001	
Low	26.8	30.8	11.0	24.0
Medium-high	73.2	69.2	89.0	76.0
**Marital status**	*p*=.397		*p*<.001	
Single mother	12.1	12.9	98.4	97.2
With partner	87.9	87.1	1.6	2.8
**Three or more siblings**	*p*=.191		*p*<.001	
Yes	13.5	14.8	4.1	6.6
No	86.5	85.2	95.9	93.4
**Family income**	*p*=.006		*p*<.001	
Lowest quintile	19.1	22.4	14.4	23.0
Second-fifth quintile	81.0	77.7	85.7	77.0

Note. Total samples (males and females combined). *p* values refer to Chi-square tests comparing percentages within Pelotas and ALSPAC.

Table S2. Prevalence of adolescent crime by four categories of childhood behaviour scores

	Conduct Problem Score					Hyperactivity Score				
	0-1	2-3	4-5	6-10	*p* value	0-1	2-3	4-5	6-10	*p* value
	% Nonviolent crime					% Nonviolent crime				
**ALL**										
Pelotas	3.9	5.9	8.4	10.8	<.001	3.0	3.4	6.7	9.1	<.001
ALSPAC	11.8	15.1	21.3	35.7	<.001	10.5	14.2	14.2	19.3	<.001
**FEMALES**										
Pelotas	1.3	2.3	5.0	5.5	<.001	1.4	1.3	3.3	4.3	<.001
ALSPAC	7.9	10.9	13.4	33.3	<.001	7.6	9.8	10.6	13.0	.021
**MALES**										
Pelotas	7.11	9.51	11.6	15.6	<.001	5.4	6.8	9.8	12.8	<.001
ALSPAC	16.6	20.7	30.1	38.5	<.001	16.2	19.1	17.6	23.5	.091

	Conduct Problem Score					Hyperactivity Score				
	0-1	2-3	4-5	6-10	*p* value	0-1	2-3	4-5	6-10	*p* value
	% Violent crime					% Violent crime				
**ALL**										
Pelotas	10.9	16.5	20.0	23.8	<.001	10.0	11.1	14.9	22.0	<.001
ALSPAC	5.0	8.9	11.6	14.3	<.001	4.3	6.6	6.2	14.0	<.001
**FEMALES**										
Pelotas	5.4	8.6	14.0	14.8	<.001	5.7	6.1	6.4	14.8	<.001
ALSPAC	2.0	4.4	2.4	13.3	.004	2.5	1.9	4.5	4.4	.091
**MALES**										
Pelotas	17.9	24.3	25.7	32.1	<.001	16.8	19.1	22.6	27.7	<.001
ALSPAC	8.7	14.8	21.9	15.4	<.001	7.8	11.8	7.8	20.6	.002

Note. *p* value for linear trend

Table S3. Associations between childhood behaviour problems and crime, adjusted for confounders, using complete cases

		PELOTAS		ALSPAC		PELOTAS-ALSPAC Interaction	
		**RR (95% CI)**	***p* value**	**RR (95% CI)**	***p* value**	**RRR (95% CI)**	***p* value**
**FEMALES**							
**Behavioural Predictor**	**Crime Outcome**						
Conduct	Nonviolent	2.7 (1.4-5.3)	.005	1.8 (0.6-1.9)	.057	1.5 (0.6-3.7)	.397
Hyperactive	Nonviolent	1.5 (0.7-3.0)	.299	1.0 (0.5-0.1)	.925	1.4 (0.5-4.3)	.556
Conduct	Violent	1.5 (1.0-2.0)	.029	1.6 (0.8-0.9)	.361	0.9 (0.6-1.3)	.601
Hyperactive	Violent	2.0 (1.4-2.8)	<.001	1.2 (1.0-0.3)	.780	1.6 (0.8-3.3)	.195
**MALES**							
**Behavioural Predictor**	**Crime Outcome**						
Conduct	Nonviolent	1.4 (1.0-1.9)	.082	1.4 (0.4-1.4)	.173	1.0 (0.5-2.0)	.921
Hyperactive	Nonviolent	1.3 (0.9-1.8)	.126	1.2 (0.3-0.9)	.390	1.0 (0.6-1.9)	.890
Conduct	Violent	1.2 (1.0-1.5)	.094	1.4 (0.2-1.7)	.082	0.9 (0.3-2.4)	.794
Hyperactive	Violent	1.3 (1.1-1.6)	.007	1.3 (0.2-1.5)	.126	1.0 (0.4-2.7)	.984

N = 1,701 females and 1,587 males in Pelotas, 1,211 females and 976 males in ALSPAC.
RR = Risk ratio adjusted for conduct and hyperactive problems age 11; unplanned pregnancy, ever smoked in pregnancy, alcohol use in pregnancy, urinary infection in pregnancy, intrauterine growth restriction, premature birth, maternal age, maternal education, marital status, 3+ siblings, low family income, parental crime birth-age11, maternal mental health age 11, child age in months at time of crime assessment
CI = Confidence Interval
RRR = Ratio of adjusted risk ratios = RR for Pelotas divided by RR for ALSPAC
